# Data linkage of Psychiatric and Maternity data to investigate the pregnancy outcomes of women with Non-affective Psychosis in Scotland

**DOI:** 10.23889/ijpds.v1i1.318

**Published:** 2017-04-19

**Authors:** Paula Mcskimming, Sarah Barry, John Park, Sohinee Bhattacharya, Angus MacBeth

**Affiliations:** Robertson Centre for Biostatistics, University of Glasgow; PsyCIS Team, NHS Greater Glasgow and Clyde; University of Aberdeen; University of Edinburgh

## Objectives

Women with a diagnosis of non-affective psychosis have a lower fertility rate than the general population. However, perinatal outcomes in mothers with non-affective psychosis are under-researched. What is the general fertility rate (GFR) of women with a lifetime diagnosis of non-affective psychosis in Scotland? Does such a diagnosis affect the outcome of pregnancy? 

## Approach

An ‘exposed’ cohort with non-affective psychosis and at least one pregnancy was established using a combined dataset derived via data linkage in local safe havens of routine psychiatric and maternity data from two Scottish regions:

- Grampian (NHSGr): Aberdeen Maternity and Neonatal Databank (AMND); psychiatric casenotes
- Greater Glasgow and Clyde (NHSGG&C): SMR02; PsyCIS bespoke psychiatric database

Exposed women were matched to women without a diagnosis of non-affective psychosis, by maternal age (NHSGG&C only), year of first birth, parity and deprivation, sourced from AMND/SMR02 in a 3:1 unexposed:exposed ratio.

Demographics and pregnancy outcomes of exposed versus unexposed women were analysed to describe effect of psychosis on pregnancy. 

## Results

Many challenges were encountered in terms of having legal agreements in place between institutions and safe havens, constructing cohorts and datasets within each study site and joining the data to analyse the overall cohort. Challenges with the data itself included discrepancies between the variables measured in datasets in different sites and missing information within patient records, particularly in earlier years.

Preliminary results for the NHSGG&C region show that the average GFR for exposed women aged 15-44 from 2005 to 2014 was 14.38 compared to the general population rate of 55.26. The number of women ever having a miscarriage was significantly higher in the exposed group (23.4% vs 9.9%; p-value <0.001). However during the study period (1996 to 2014), more unexposed women had miscarriages (0.5% vs 4.7%; p-value = 0.002). There were no significant differences in pregnancy complications for the study period. The mean birthweight of babies was lower (3.23kg vs 3.35kg; p-value = 0.029) and more babies were admitted to neonatal units (17.5% vs 9.8%; p-value = 0.004) in the exposed group.

Results for the NHSGr region and the combined dataset will also be reported.

## Conclusions

This work highlights that there remain hurdles to linking data across sites, despite availability of rich datasets within Scotland. Women with non-affective psychosis within NHSGG&C region had a lower fertility rate on average than the general population and some poorer outcomes, such as birthweight and rate of admission to neonatal units.

